# Factors Affecting Nurses’ Coping With Transition: An Exploratory Qualitative Study

**DOI:** 10.5539/gjhs.v6n6p88

**Published:** 2014-07-15

**Authors:** Jalil Azimian, Reza Negarandeh, Ali Fakhr- Movahedi

**Affiliations:** 1 School of Nursing and Midwifery, Tehran University of Medical Sciences, Iran; 2 School of Nursing and Midwifery, Nursing and Midwifery Care Research Center, Tehran University of Medical Sciences, Iran; 3 School of Nursing and Midwifery, Semnan University of Medical Sciences, Iran

**Keywords:** transition, coping, nurse, qualitative study

## Abstract

**Aim:**

One of the most important factors contributing to staff shortage is nurses’ ineffective coping with transitions. Changes in nurses’ official positions are usually associated with varying degrees of transition. Identification of affecting factors on nurses’ coping in responding to transition can promote quality of nursing activity and prevent nurses’ shortage. So the aim of this study was to explore factors affecting nurses’ coping with transitions.

**Methods:**

The participant of this exploratory qualitative study consisted of sixteen nurses that were work in medical wards of four hospitals in Qazvin, Iran. Data collected by semi-structured interviews. The data were analyzed by qualitative content analysis approach.

**Results:**

The main theme of the study was ‘inadequate preparation for transition’. This theme consisted of six categories including “staff training and development”, “professional relationships”, “perceived level of support”, “professional accountability and commitment”, “welfare services”, and “nursing staff shortage”.

**Conclusion:**

Nursing managers and policy makers need to pay special attention to the affecting factors on nurses’ coping with transition and develop effective strategies for facilitating it.

## 1. Introduction

In recent years, many countries have faced serious nursing staff shortage (Buchan & Calman, 2004; Buerhaus et al., 2009; Leong, 2009; Salera-Vieira, 2009; Hollywood, 2011). Currently, nursing staff shortage is a major healthcare challenge worldwide (Buerhaus et al., 2009). The same problem also exists in our country, Iran. Although the number of hospital beds has increased significantly in Iran during recent years, the number of nursing staffs has precipitated the crisis of staff shortage (Farsi et al., 2010), which has precipitated the crisis of staff shortage (Zarea et al., 2009; Farsi et al., 2010). Many factors such as hospital managerial style, working in long shifts, nurses’ heavy workload, and ineffective coping with transitions contribute to this crisis (Rahimi et al., 2004; Cowin & Hengstberger-Sims, 2006; Salt et al., 2008).

Transition is defined as the process of moving from a state to another one which is usually associated with significant changes in goals, roles, and responsibilities (Oplatka, 2001; Duchscher, 2009; Meleis, 2010). Studies have shown that during transition, different problems such as anxiety, tension, fear, panic, and burnout endanger and undermine nurses’ physical and mental health (Kelly & Mathews, 2001; Zerwekh & Claborn, 2006; Duchscher, 2009). These problems accelerate staff turnover rate and result in the replacement of experienced nurses by novice ones who do not have enough knowledge, experience, and confidence for working in clinical settings (Duchscher, 2009). Given the direct relationship between nurses’ quality of working life and the quality of care provided by them, the final outcome of ineffective coping with transition would be inferior quality of nursing care and negative patient outcomes (Mohammadi et al., 2011).

Effective coping with transition-related stress and anxiety is extremely important to individual’s life and development (Williams, 1999). Inattention to factors affecting nurses’ coping with transition would negatively affect their performance, undermine their effectiveness, and result in isolation, overdependence, fear, denial, projection, job dissatisfaction, poor job motivation, inability to fulfill professional responsibilities, and paying attention only to technical aspects of care instead of providing care based on critical thinking (Fugate et al., 2002; Badger, 2005; Marks, 2007). It may finally endanger different aspects of nurses’ lives as well as healthcare organizations (Manning & Neville, 2009).

The aim of this study was to explore factors affecting nurses’ coping with transitions. This was an exploratory qualitative study. The study sample consisted of sixteen nurses working in medical wards of four hospitals located in Qazvin, Iran.

### 1.1 Background in Iran

Basic nursing education in Iran is at the baccalaureate degree (Farsi et al., 2010). Currently, 90000 staffs practice nursing in Iran (Zarea et al., 2009). However, due to the increasing number of hospital beds during recent years, it is estimated that about 220000 nurses are needed for providing nursing care to Iranian clients (Zamanzadeh & Seyed, 2006; Zarea et al., 2009). Accordingly, like many countries, Iranian system of nursing care delivery is also suffering from a serious nursing staff shortage (Farsi et al., 2010; Borhani et al., 2012).

Given the close association between the effectiveness of nurses’ coping with transitions and their intention to leave the profession, identifying factors that affect transition assumes an added importance. However, despite its fundamental importance, little attention has been paid to Iranian nurses’ coping with transition (Abedi et al., 2004; Manning & Neville, 2009). The aim of this study was to explore factors affecting nurses’ coping with transitions.

## 2. Methods

Participants selected purposively and recruited from both genders—five male and eleven female nurses—and also from both bachelor’s (fourteen nurses) and master’s degrees (two nurses). Data collection In this study, the study data were collected by conducting semi-structured personal interviews. Interview questions were initially open-ended and guided by some cues such as problems that nurses did experience, nurses’ coping strategies and other influencing factors on their coping. Interviews were arranged according to participants’ preferences. All the interviews were conducted at the end of participants’ working shifts, in a quiet room located in their ward. Interviews ranged in length from 28 to 87 minutes and all of them recorded by digital sound recorder. Data collection was continued until reaching data saturation.

### 2.1 Data Analysis

Data analysis was conducted coincidently with data collection. We used the qualitative latent content analysis (Graneheim and Lundman’s approach) for data analysis (Graneheim & Lundman, 2004).

In the first, each interview transcribed verbatim. Then whole interview was considered as the units of analysis. The meaning units (i.e. words, sentences, and paragraphs) were identified, condensed, and coded. Generated codes were then compared and categorized according to their differences and similarities. Finally, categories were compared and included in the main theme of the study.The main theme stood for the latent content of the interviews. The data managed by MAXQDA 10software. We used Guba and Lincoln’s criteria for ensuring the trust worthiness of the study findings. Member check and peer check were done for credibility of data analysis.

### 2.2 Ethical Considerations

This study was approved by the Ethics Committee of Tehran University of Medical Sciences, Tehran, Iran.

We explained the aim of the study to the participants and asked them to read and sign the informed consent form. Moreover, we ensured them that their personal information would be analyzed and reported confidentially and anonymously. Participants were free to participate in or withdraw from the study.

## 3. Results

The main theme of the study was ‘Inadequate preparation for transition’. Most of our participants highlighted that they had not been adequately prepared for transition. This responsibility was delegated to me so suddenly that I became completely stunned (Participant 11). As I was not adequately prepared, it was very difficult to me; I became completely confused (Participant 3). Because of inadequate preparation, we even couldn’t manage equipment alarms (Participant 9). This main theme comprised six categories including staff training and development, professional relationships, perceived level of support, professional accountability and commitment, welfare services, and nursing staff shortage (Table 1). We explain these categories in what follows. Staff training and development Staff training and development play an important role in preparing nurses for transition. Two participants who had received trainings and preparations, were more capable of coping with transition-related strains and tensions. Most of our participants emphasized the great importance of education and training in developing coping skills. Training has been extremely helpful for me in coping with transition. [Because of trainings,] I have not experienced any insoluble problem so far (Participant15). The orientation course really helped me get prepared and cope with transition (Participant 14). However, most of participants reported that they had not been prepared for transition. A major factor contributing to their unpreparedness for transition was the inefficiency of university system.

**Table 1:** Identified factors affecting nurses’ coping with transition: Themes, categories and codes and Sub-categories

Sub-categories	Categories	Theme
Inadequate preparation due to ineffectiveness of university educations		
The role of effective university educations in preparing nurses for transition		
The role of in-service theoretical and practical trainings and orientation courses in preparing nurses for transition	Staff training and development	
The availability of qualified mentors and preceptors		
Ineffective educations about establishing professional relationships		
The role of effective communication with authorities in nurses’ coping with transition		
The role of effective communication with experienced nurses in coping with transition	Professional relationships	
The role of effective communication with peer nurses in coping with transition		
The availability of support systems during transition		
The role of physicians’ support in nurses’ coping with transition	Perceived level of support	Inadequate preparation for transition
The role of peer support in preparing nurses for transition		
The role of authorities’ support in preparing nurses for transition		
The role of family support in nurses’ coping with transition		
Nurses’ accountability for getting prepared	Professional accountability and commitment	
Nurses’ commitment as a determining factor in self-learning		
The role of welfare services in nurses’ coping with transition	Welfare services	
Lack of welfare services as a barrier to coping		
Severe staff shortage as a barrier to coping		
Adequate number of staff nurses as a facilitator to coping	Nursing staff shortage	
Heavy workload (because of staff shortage) as a barrier to coping		

Most of our participants were dissatisfied with their academic theoretical and practical trainings and referred to such inadequate trainings as an important factor contributing to their unsuccessful transition. University educations were mostly textbook-bound. Educations were not practical and did not prepare us for clinical practice (Participant 13). Our internship program was of low quality. It did not prepare me for the current practice (Participant 9). Besides lack of effective university education, most of our participants also referred to the inadequacy of on-the-job trainings as an important factor contributing to their poor preparation for transition. When I graduated and entered clinical practice, they also did not provide us with in-service trainings (Participant13). About one year has passed since they have designated us for clinical practice. However, we have not received any training since then and have not been prepared for the new responsibility (Participant 4). Unavailability of qualified preceptors and mentors a main sub-category of the staff training and development category was the unavailability of qualified preceptors and mentors. In the Iranian healthcare system, no official position is secured for mentors and preceptors. Accordingly, most nurses, during transition, tend to select experienced, qualified colleagues as role models and strive to learn their educations and behaviors.

The process of role model selection is, however, completely individual, accidental, and unplanned. Sometimes we seek advice from more experienced colleagues (Participant 15). Well, most of them are more experienced than us. Their knowledge and experiences are very helpful to us. I usually try to use their knowledge and experiences (Participant 6). However, most of our participants referred to the unavailability of qualified preceptors and mentors as a barrier to their coping with transition. After entering clinical practice, sometimes I needed someone [experienced] to help me become experienced. However, there was no such a person (Participant 15). Friendly relationships having good communication skills for establishing friendly relationships with physicians, nurses, and other colleagues was a major facilitator to our participants’ coping with transition. I have good social relationships. I have had no problem with anyone [so far]. I have always tried to establish healthy relationships with others. As a result, I was able to cope easily (Participant 16). I think that the main reason for my easy coping [with transition] was that I established friendly relationships with physicians and experienced nurses. These [friendly relationships] greatly helped me get prepared for coping with difficulties (Participant 16).

Although having good communication skills facilitated coping with transition, our participants noted that there was an apparent lack of strong relationships between nurses and other care providers. I have not been able to establish good relationships with other colleagues so far. Consequently, after passing eighteen months of my employment, I have not coped yet (Participant 9). Probably a major barrier to establishing relationship with other care providers was the routine-oriented organizational culture. A nurse needs to be vigilant and cautious about establishing relationships. He/she needs to avoid saying anything that is irrelevant to work. I’ve strived to avoid entering debates (Participant 8). Perceived level of support during transition, nurses feel a great need for support. Accordingly, the support given by managers, experienced nurses and colleagues, and nurses’ families significantly contributed to our participants’ coping with transition. Besides managers, physicians, and my own colleagues also considerably helped me cope [with transition] (Participant 15). Nursing is a depressing job. In our job, we are constantly in touch with illness and stuffs like that. Accordingly, family support is a matter of great importance. The unavailability of family support would either slow down or disrupt nurses’ coping (Participant 16). One of our participants who had effectively coped with transition after nine months also referred to the support of her family as the main reason for her effective coping. My family members have always supported me. Accordingly, I had no difficulty in coping [with transition] in such a crowded ward (Participant 6). Conversely, lack of family support during transition—while nurses are under heavy strains—was a major barrier to their coping with transition. One participant who suffered from severe work-related stress and needed her spouse’s support for coping with the situation explained her experience of getting no support from her spouse as follows. My husband just says, ‘Either change your job or keep silent’. He never supports me (Participant 14). Most of our participants noted that nurses usually do not receive adequate support from their managers, colleagues, peers, and friends during transition. I think that our nursing system is the worst system in the country. It does not support its nursing staffs … it never supports (Participant 15).

Professional accountability and commitment according to our participants, professional accountability and commitment motivate nurses to gain more knowledge and experience and to get adequately prepared for transition. They noted that nurses who are more accountable and more committed feel greater responsibility towards self-learning, participating in continuing education programs, and interacting with physicians and other colleagues and hence, are able to cope with transition. I decided and strived to be the best wherever I am. Accordingly, I easily coped with transition (Participant 3). I was highly motivated; I always liked learning; I worked hard and studied related textbooks. Accordingly, I coped rapidly (Participant 14). When I entered the [new] ward, I usually studied the textbooks. In other words, I learned on my own. There was nobody to teach me. I could cope easily because I tried hard (Participant 9). [Sometimes] I woke up [early] at 05:30 A.M and studied. I don’t like to perform a job without having enough knowledge. I like to have information and to perform my duties flawlessly. As a result, I easily coped with the difficulties of transition (Participant 8) Welfare services.The availability of adequate rest and refreshment facilities was another factor affecting our participants’ coping with transition. Some participants noted that during their transition, their organization had provided them with some sort of such facilities. Our nursing office was very helpful. They [nurse managers] provided us with whatever we needed and resolved our problems (Participant 7). However, according to most of our participants, serious lack of welfare services for nurses was a major barrier to their coping with transition. There is neither nursery nor transportation facilities here [in the hospital]. They provide [us with] nothing. They do not provide us with dinner; therefore, we have to either starve till morning or take dinner from home. Moreover, the availability of [welfare] services and facilities is being reduced day by day. I’m not interested in nursing anymore. I’m just waiting for completing the current work assignment. Afterward, I will surely leave nursing forever (Participant 14). Currently, there is a general shortage of welfare facilities for nurses in the Iranian healthcare settings. Accordingly, the limited amount of facilities and resources is mainly allocated to more experienced, senior nurses. More experienced ones had the choice to take rest in cozy places. However, we [the juniors] had no option but to find a place in basement or cellar to rest (Participant 6). Staff shortage According to our participants, severe nursing staff shortage was one of the most important factors contributing to nurses’ ineffective coping with transition.

Most of our participants had been required to work on multiple long shifts or during holidays because of staff shortage. Some of our participants had even worked in continuous and long shifts for 48 to 72 hour shifts. The consequences of such obligatory shifts were increased workload, physical and mental fatigue, and ineffective coping with transition. After a night shift, I had to work on a morning-evening one. [Such shifts] made me extremely tired. I was completely exhausted (Participant 10). Because of staff shortage, we were heavily involved in work and terribly busy. [We worked] multiple shifts morning, evening, and night. I was always tired [and therefore,] decided to leave nursing and apply for a new job with lighter workload (Participant 14). On the other hand, because of heavy staff shortage, orientation courses were too short and ineffective, if there were any. Accordingly, nurses entered the transition phase without any effective orientation and prior preparation. University educations are limited and [hence,] when I began clinical practice, I had so much stress that I didn’t know what I had to do with patients (Participant 13). Poor nurse; there is no orientation or preparation courses. Therefore, it [beginning the clinical work] is like being thrown in a stormy sea. It takes a great deal of time [for a nurse] to be capable of finding his way [in such a sea].

## 4. Discussion

The aim of this study was to explore factors affecting nurses’ coping with transitions. Study findings revealed that factors such as staff training and development, professional relationships, perceived level of support, professional accountability and commitment, welfare services, and nursing staff shortage potentially contribute to nurses’ coping with transitions. These factors were included in a broad theme entitled, ‘Inadequate preparation for transition’. Accordingly, the major barrier to our participants’ coping with transition was their inadequate preparation for it. Meleis (2010) noted that adequate preparation facilitates the process of transition while lack of preparation is a hindrance to it. Preparation fundamentally deals with acquiring the basic knowledge of transition-related incidents and effective strategies for managing them (Meleis, 2010). However, our findings indicated the ineffectiveness of university educations in preparing nurses for transition. Adib-hajbagheri et al. (2004 and 2005) reported that university-based nursing educations in Iran not only fail to adequately prepare nursing students for transition but also reduce their professional autonomy (Adib Hajbaghery & Salsali, 2005). Moreover, despite the known effects of orientation on transition management (Deppolitti, 2003; Duchscher, 2009; Meleis, 2010), and (Xu & He, 2012), we found that lack of effective orientation courses was a barrier to our participants’ preparation for transition. Similarly, Xu and He (2012) reported that orientation programs are not routinely conducted in the United States. Nurses’ inadequate preparation for transition is associated with negative outcomes such as high staff turnover and subsequent exacerbation of nursing staff shortage (Xu & He, 2012).

We also found that the unavailability of qualified preceptors and mentors contributed to nurses’ inadequate preparation for transition. Thomes (2003) noted that the availability of qualified preceptors and mentors could help novice staffs effectively cope with transition-related problems and difficulties (Thomes, 2003). Consequently, providing nurses with preceptor- and mentor-mediated trainings would be an effective strategy for improving their coping skills and preparing them for transitions. Another factor affecting our nurses’ coping with transition was poor professional relationships. Hutchinson et al. (2006) also reported that communication problems cause nurses to resort to ineffective defense mechanisms such as anger, isolation, and silence which in turn aggravate the situation and slow down their coping with transition (Hutchinson, Vickers, Jackson, & Wilkes, 2006). The study findings also revealed that the level of perceived support was an important factor in nurses’ coping with transition. Generally, inadequate support for nurses during transition is associated with burnout and job dissatisfaction (Laschinger, Finegan, & Wilk, 2009; Patrick & Laschinger, 2006). Dyess and Parker (2012) reported that educational support had positive effects on nurses’ professional skills, retention in the profession, and coping with transition (Dyess & Parker, 2012). Studies have shown that peers, families, spouse, and parents are the main sources of emotional support for nurses (Lo, 2002). Financial support can also help novice nurses effectively cope with transition-related difficulties (Lo, 2002; Walker, 2008). Accordingly, nurse managers can help nurses cope with transitions by providing them with different types of support.

We also found that nurses’ professional accountability and commitment were another factors affecting their coping with transition. Currently, organizations strive for recruiting highly committed problem-solver employees who can efficiently use their knowledge and expertise for enhancing organizational effectiveness (Duchscher, 2009). Nonetheless, healthcare managers and policy makers have taken professional commitment for granted (Adib Hajbaghery & Salsali, 2005). Another factor affecting our participants’ coping with transition was serious nursing staff shortage. This finding is in line with the findings of other studies (Duchscher, 2009; Hearnden, 2008; Patrick & Laschinger, 2006). Ineffective coping with transition because of staff shortage, in turn, significantly contribute to nurses’ intention to leave the profession (Reising, 2002). This vicious cycle unceasingly exacerbates both problems—i.e. nursing staff shortage and ineffective coping with transition. Accordingly, identifying transition-related sources of occupational stress can help nurse managers develop effective strategies for promoting staff retention (Dyess & Parker, 2012). Finally, we found that serious lack of welfare services for nurses was a major barrier to their coping with transition. Other studies conducted in our country have also reported the same finding (Abedi, Heidari, & Salsali, 2004; Adib Hajbaghery & Salsali, 2005). However, this finding contradicts the findings of studies conducted in other countries (Deppolitti, 2003; Duchscher, 2009; Laschinger, Finegan, & Wilk, 2009; Meleis, 2010; Patrick & Laschinger, 2006).

## 5. Conclusion

This study explored factors affecting nurses’ coping with transitions. Study findings indicate that transition is a difficult period in nurses’ working life during which they need the strong support from their families, peers, and managers. The major factor affecting nurses’ coping with transition is their preparedness. Factors such as staff training and development, professional relationships, perceived level of support, professional accountability and commitment, welfare services, and nursing staff shortage contributes to nurses’ preparation for transition. Given the paramount importance of transition, nurse managers and policy makers need to pay special attention to these factors and. to develop effective strategies for removing barriers and facilitating nurses’ coping with transition. Accordingly, developing and implementing comprehensive staff development and training programs, initiating mentoring programs, strengthening nurses’ professional relationships, supporting nurses during transitions, promoting their professional accountability and commitment, and enhancing the effectiveness of management systems are recommended for facilitating nurses’ coping with transitions. Further studies are still needed for producing ample evidence regarding nurses’ coping with transitions. Accordingly, exploring the process of coping with transitions as well as the facilitators and barriers to transitions in different contexts is recommended.
